# Occupational Therapy in HomEcare Re-ablement Services (OTHERS): study protocol for a randomized controlled trial

**DOI:** 10.1186/1745-6215-15-447

**Published:** 2014-11-19

**Authors:** Phillip J Whitehead, Avril ER Drummond, Marion F Walker, Ruth H Parry, Ian D McGeorge, Zaid Latif

**Affiliations:** Division of Rehabilitation and Ageing, University of Nottingham, The Medical School, Queens Medical Centre, B Floor, Derby Road, Nottingham, NG7 2UH UK; School of Health Sciences, University of Nottingham, The Medical School, Queens Medical Centre, A Floor, Derby Road, Nottingham, NG7 2HA UK; c/o Division of Rehabilitation and Ageing, University of Nottingham, B Floor, The Medical School, Queens Medical Centre, Derby Road, Nottingham, NG7 2UH UK; Nottingham City Council, Adult Social Care and Health, Mary Potter Centre, Gregory Boulevard, Nottingham, NG7 5HY UK

**Keywords:** Occupational therapy, Homecare re-ablement services, Activities of daily living

## Abstract

**Background:**

Homecare re-ablement services have been developed by local authorities in England in response to the government agenda for health and social care. These services aim to optimize users’ independence and ability to cope at home, and reduce the need for ongoing health and social care services. However, there is currently limited evidence and guidance regarding the optimum configuration and delivery of re-ablement services. In particular, the impact of occupational therapy input on service user outcomes has been highlighted as a specific research priority.

**Methods/Design:**

This feasibility randomized controlled trial (RCT) will recruit 50 people from one local authority led homecare re-ablement service in England. Those who provide informed consent will be randomized to receive either usual homecare re-ablement (without routine occupational therapy input) or usual homecare re-ablement plus an enhanced program targeted at activities of daily living (ADL), delivered by an occupational therapist. The primary aim of this study is to assess the feasibility of conducting a further, powered study. The participant outcomes assessed will be independence in personal and extended ADL, health and social care-related quality of life, number of care support hours, falls, acute and residential admissions and use of health and social care services. These will be assessed at two weeks, three months and six months post-discharge from the re-ablement service.

**Discussion:**

To our knowledge, this is the first RCT of occupational therapy in homecare re-ablement services. The results of this study will lay the foundations for a further powered study. The findings will be relevant to researchers, clinicians, commissioners and users of adult social care services.

**Trial registration:**

Current Controlled Trials registration number: ISRCTN21710246 (registered on 31March 2014)

## Background

In England, the Department of Health has highlighted its commitment to re-ablement services in the white paper *Caring for Our Future: Reforming Care and Support* [1] *.* This document outlines the government’s vision that the provision of these services should support people to remain living independently in their homes after a crisis event. It has been further highlighted that the National Health Service (NHS) and local authorities should work jointly to provide support for people leaving hospital, or recovering from illness or injury, in order to improve outcomes for users (such as their ability to manage personal care independently), and to deliver cost savings for both organizations [2]. However, there is limited guidance regarding the optimum configuration and skill mix of re-ablement teams [3], and the role and remit of occupational therapists in these services has been highlighted as a particular research priority [4].

### ‘Homecare’ and ‘homecare re-ablement services’

Within adult social care services in the United Kingdom, ‘homecare’ is the term used to describe a type of community service in which a care worker visits the person at home to give assistance with everyday activities such as washing, dressing and meal preparation. Homecare is usually delivered by care workers who may or may not have a vocational care qualification, and does not usually involve qualified healthcare professionals. Homecare may be provided on a short- or long-term basis, depending on the needs of each individual service user [5].

In a homecare re-ablement service, users receive homecare but are supported to increase their ability to manage tasks independently, in order to reduce the amount of homecare they will require in the longer term [6]. Increasingly, such services have been developed by local authorities in England to work with people who are newly referred to social care services as needing homecare support [3, 7]. This could mean adults having difficulties managing independently at home, for example, people leaving hospital, recovering from illness or injury or experiencing a new deterioration of a long-term condition. Homecare re-ablement teams usually work with users for up to six weeks, with some flexibility to extend this in certain circumstances [8]. After six weeks, those users who require ongoing help are referred to a traditional homecare team for longer-term support. Internationally, comparable services are referred to as ‘restorative homecare’ [9–12], and may operate within similar parameters to homecare re-ablement.

Homecare re-ablement differs from community rehabilitation in that services adopt a social model of recovery rather than a medical model [13]; there is also a specific focus on reducing the need for paid homecare support. Whilst some re-ablement teams have significant therapy input, others are managed and staffed by social care workers who may have received training in re-ablement philosophies and approaches [6].

### Homecare re-ablement and occupational therapy

Eligibility for homecare re-ablement services is predominantly based around the need for assistance with personal care activities in the home environment. Increasing independence in activities of daily living (ADL), and modifying the home environment to increase independence and reduce risks, are among the core skills of the occupational therapist [14]. These skills are clearly compatible with homecare re-ablement service aims and this has been identified by the United Kingdom College of Occupational Therapists (COT). In a position statement published in 2010, the COT highlighted the similarities between the philosophy of occupational therapy and re-ablement approaches, and argued that successful outcomes for service users and demonstrable cost benefit for local authorities depended upon the involvement of occupational therapists [15].

Previous studies have suggested that targeted occupational therapy assessment and intervention can improve people’s ability to manage ADL independently in a variety of different contexts [16–20]. However, specific research is required within re-ablement services in order to test the effectiveness with this particular service user group, evaluate the impact on users’ ability to manage independently and assess the effects on the cost of ongoing health and social care services.

### Why is this study needed?

Homecare re-ablement services are currently high on the policy agenda, forming part of a national strategy to reduce the number of people requiring ongoing care [1]. The primary goal of these services is to assist users to regain lost skills and abilities [21], with an emphasis on those skills pertaining to personal care and meal preparation. Given the specialist skills of occupational therapists in this area, it may seem logical that occupational therapists should be involved in these services; however, the extent and nature of their input varies nationally and internationally. Services have developed sporadically across England, in line with regional variations in health and social care commissioning policies, and thus not all teams directly incorporate the skills of occupational therapists [22].

A controlled before and after study published in 2010 compared five local authority sites with re-ablement services with traditional homecare services [8]. This study reported that 29% of the users who received the re-ablement service had input from an occupational therapist during the re-ablement episode. However, the study authors reported that it was not possible to determine whether the outcomes were better for users who received occupational therapy input compared to those who did not [8]. Thus, there is a requirement for further research which investigates outcomes for service users who receive occupational therapy. Research should also examine whether there are cost savings for local authorities associated with the inclusion of such a targeted occupational therapy intervention in homecare re-ablement teams.

As a precursor to this randomized controlled trial (RCT), a systematic review was conducted. The protocol was published prospectively [23] and the manuscript is currently in preparation. No previous RCTs of occupational therapy interventions for users of homecare re-ablement or restorative homecare services were identified in the systematic review.

## Methods/Design

### Research aim and objectives

Our overall aim is to conduct an RCT to determine whether an occupational therapy intervention can improve the ability of homecare re-ablement service users to carry out ADL independently. This feasibility RCT will enable us to ascertain the viability of conducting a definitive appropriately powered study. It will also allow us to conduct preparatory health economic evaluation work.

### Study setting

This single-centre feasibility study will be conducted within one city council homecare re-ablement service in England. The service is divided into six geographical sub-teams. This RCT will be conducted within one sub-team, which currently does not have routine input from an occupational therapist.

### Participants

The re-ablement service accepts referrals from any adult aged over 18 years, living in the community, with a need for homecare support, except those with a diagnosis of dementia who have a specialist dementia homecare service within the area. All users of the homecare re-ablement service within the selected sub-team will be screened for eligibility. Inclusion criterion for the trial is the ability to provide informed written consent. Exclusion criteria are: inability to speak English, on an end-of-life care pathway, requiring assistance from two or more people to transfer or receiving input from a community rehabilitation team.

### Intervention and comparator

Participants will be randomized to either usual homecare re-ablement (control group) or the occupational therapy intervention (intervention group). Those randomized to the control group will receive the usual routine care provided by the homecare re-ablement service, that is, a six-week period of homecare re-ablement provided by re-ablement workers (paid care workers), under the direction of a re-ablement care team leader. This does not involve input from qualified health professionals. Therefore, the control group will not routinely receive specialist ADL assessment and intervention, or routinely access community equipment or minor adaptations provided by an occupational therapist as part of their re-ablement package. This is current practice within the sub-team at the trial site. However, if service users in the control group are identified as requiring specific occupational therapy input, they will be referred to the mainstream community occupational therapy team (waiting time currently exceeds the six-week re-ablement period). Referrals to occupational therapy are not routinely made.

Those randomized to the intervention group will receive all routine homecare re-ablement services but, in addition, will receive an enhanced program targeted at ADL, delivered by an occupational therapist. The aim of the enhanced program will be to maximize independence in ADL activities including (but not limited to): washing, dressing, bathing and showering, feeding, indoor mobility, transfers, stair mobility, toileting, meal preparation and kitchen activities, outdoor mobility and community access (as appropriate to individual participants).

Following the re-ablement service referral, a systematic assessment will be completed by an occupational therapist within five working days. This will identify difficulties the service user may have with ADL due to physical impairments, psychological difficulties or a combination of both. The assessment will be used as a basis to set one or more ADL-related goals for the re-ablement episode. A program will then be agreed with the participant, which will be tailored to the needs of each individual, but will include: practicing activities, and/or a graded process of re-learning and building the skills to manage ADL independently; equipment provision and environmental or activity modification. A case management approach will be adopted by the occupational therapist involving a minimum of weekly reviews and the coordination of the re-ablement episode and other services. Advice and information will also be provided to family members or carers.

The intervention will utilize the occupational therapist’s core skills in activity analysis, where an activity is broken down into its distinct component parts in order to identify the specific element(s) of the task which the individual is unable to perform [24]. The occupational therapist will combine their medical knowledge of prognosis with their assessment of functional ability in order to select an appropriate approach for the re-ablement episode (for example a compensatory or a bio-mechanical approach).

Timely provision of community equipment and/or minor adaptations (such as grab rails, half-steps or threshold removal or replacements) will form a core component of this intervention. These will be prescribed by the occupational therapist and provided by the Community Equipment Service for the local area. The occupational therapist will complete an ongoing review of the use of equipment in order to assess whether the continued use is warranted or no longer required. The enhanced input will continue for the duration of the re-ablement episode (up to six weeks), and will cease when the service user is discharged from the re-ablement service.

### Cost evaluation

As part of the cost evaluation, a record will be kept of the number of times the occupational therapist visited each service user in the intervention group, the amount of time spent per visit and a log of what was carried out on each visit (in the form of a coded checklist). In addition, a record will be kept of the cost of equipment and minor adaptation services provided. Participants in both groups will report their use of health and social care services during the intervention and follow-up period.

### Concomitant treatments

There are no known issues with the treatment and concomitant treatments. However, participants who are receiving input from specialist community rehabilitation services (such as intermediate care or community stroke team) will be excluded. Information will be kept on the participant’s use of other community rehabilitation services (such as private therapists) and will be reviewed as part of the assessment of feasibility.

### Compliance

Compliance with the treatment intervention will be monitored by the occupational therapist delivering the intervention. This will be defined as participation in treatment visits and willingness to continue with treatment (further visits).

### Outcomes

The primary trial outcome will be a determination of the feasibility of conducting a larger, appropriately powered trial. The assessment of feasibility will be a composite measure of recruitment, retention, acceptability and the viability of delivering the intervention. Key aspects to be addressed are: whether the eligibility criteria are realistic, whether service users are willing to be randomized, the dropout rate, the content and scheduling of the occupational therapy treatment visits, the acceptability of the occupational therapy intervention, the most suitable primary outcome measure for the main study and the feasibility of the cost data collection.

**Figure 1:**
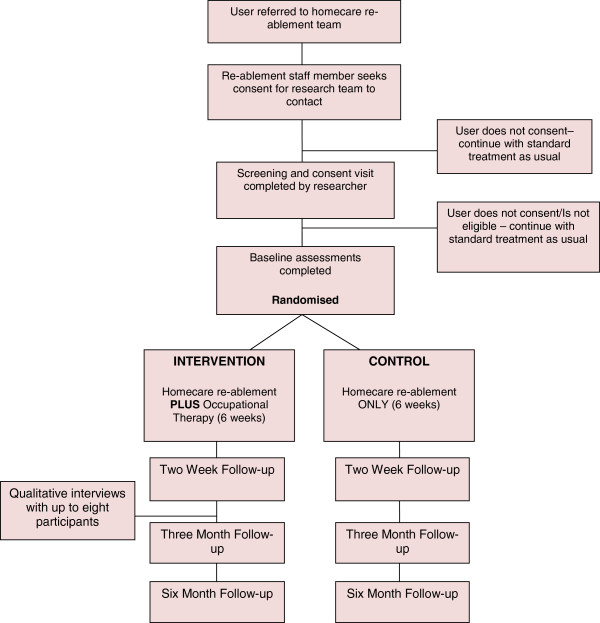
**Flow of participants through the study.**

The participant outcomes to be assessed will be: personal and extended ADL, health and social care-related quality of life, number of care support hours, health and social care service usage and carer strain. The measures are: Barthel Index (BI), Nottingham Extended Activities of Daily Living (NEADL), Short-Form 36 (SF-36), Adult Social Care Outcomes Toolkit (ASCOT), EQ-5D and Caregiver Strain Index (CSI). Additionally, information will be collected on: number of homecare hours, falls, admissions (to acute and residential services) and use of health and community services. The Mini-Mental State Examination will be completed at the baseline time point in order to provide a description of the sample. The timeline and proposed flow of participants through the study are shown in Figure 1.

Acceptability of treatment will be ascertained using a purposely designed questionnaire, which will be left with all participants in the intervention group, after the occupational therapy input has ended. This questionnaire will include questions on their views of the occupational therapy intervention and will cover its content, frequency, delivery, timing and intensity. Additionally, interviews will be completed with up to five participants who received the treatment intervention, in order to explore their views and experiences of the intervention. These post-intervention interviewees will be purposively selected for a variety of features including: underlying diagnostic condition, prior experience of receiving occupational therapy and homecare, age, those who live alone and those who live with support.

### Sample size and recruitment strategy

For a feasibility study, no formal sample size calculation is required. The aim is to recruit 50 participants (25 in each arm of the trial) to test the randomization process and the acceptability of the intervention. The trial will recruit for eight months. Current data from the trial site suggests that approximately three service users per week will be eligible. A maximum of 10 participants will be recruited per calendar month. Potential participants will be approached in the order in which they are referred to the re-ablement service. If 10 participants consent during a calendar month, recruitment will cease until the first new referral of the next calendar month. If the maximum of 50 participants are recruited before the end of the eight-month period, recruitment will cease.

Participants will be enrolled into the study by the investigator. The process for obtaining informed consent will be in accordance with the Research Ethics Committee (REC) guidance, and Good Clinical Practice (GCP) and any other regulatory requirements that might be introduced. The investigator and the participant shall both sign and date the informed consent form before the person can participate in the study. Randomization will be generated online by the Nottingham Clinical Trials Unit (NCTU) using a web-based randomization program. Participants will be individually randomized in random varying block sizes (sized in order to deliver the clinical intervention appropriately). Randomization will be at a ratio of 1:1 (treatment-to-control). Only the NCTU will have access to the allocation sequence. Baseline assessments will be completed prior to randomization. Follow-up assessment visits will be completed by a research assistant who is masked to the treatment allocation. It is possible that participants may reveal their treatment allocation to this assessor, and any instances of this will be recorded as part of the assessment of feasibility.

### Data collection, management and analysis

Data will be collected in the participants’ homes on a paper case report form (CRF) and will subsequently be entered onto a secure password-protected electronic database. Outcome data will be entered by the research assistant who collected the data (and thus will be entered blind to treatment allocation). Each participant will be assigned a trial identity code number, allocated at randomization, for use on CRFs, other trial documents and the electronic database. The documents and database will also use their initials and date of birth.

CRFs will be treated as confidential documents and held securely in accordance with regulations. The investigator will make a separate confidential record of the participant’s name, date of birth, local social care number and participant trial number to permit identification of all participants enrolled in the trial, in accordance with regulatory requirements, and for follow-up assessments as required. Access to CRFs shall be restricted to those personnel approved by the chief investigator.

When data collection is complete, a data quality check will be conducted in duplicate by two researchers and a 10% sample of the database will be checked against the original paper CRF. Steps will be taken to minimize missing data via personal contacts throughout the study period from the investigator, and every attempt will be made to locate participants for follow-up assessment. Outcome data will be collected in person by a research assistant to minimize the amount of missing data. For each outcome measure used where data is missing, an imputed average will be used for items where less than 10% of the overall measure is missing. Where more than 10% of a measure is missing, the entire measure will be coded as missing.

The main endpoint for the study is determination of the feasibility of conducting a larger, powered study. Descriptive statistics will be used for this analysis, based on analysis of the trial screening and recruitment log, loss to follow-up and analysis of the acceptability questionnaire. Analysis of outcome data will be by the intention-to-treat principle, and participants will be analyzed according to their treatment assignment irrespective of whether they completed the treatment. It will not be possible to collect any outcome data for those who discontinue participation in the study. The data collected from the outcome measures in the trial will be presented using summary statistics, and any differences between the arms will be calculated at two-week, three-month and six-month follow-up assessments, along with the 95% confidence intervals. This data will be used to inform a sample size calculation, treatment effect estimate and to determine the appropriateness of these measures for use in a larger, powered study. Assistance from a statistician will be available as required. A health economist will provide input for the economic evaluation of the intervention and analysis of the EQ-5D data.

A Trial Steering Committee (TSC) is in place and includes experienced health and social care researchers, social care staff, an experienced geriatrician and public and patient representatives. Ethical approval for this study was provided by The Social Care Research Ethics Committee (approval number: 13/IEC08/002), and management approval has been obtained from the trial site.

### Safety monitoring and adverse events

We are not anticipating any adverse events as part of this intervention; however, we will monitor adverse events during the course of the study. Participants will be asked about any adverse events (including hospital admissions and falls) during all treatment and follow-up visits. All serious adverse events will be recorded and closely monitored until resolution, stabilization or until it has been shown that the study treatment or intervention is not the cause. One investigator (AD) will be informed immediately of any serious adverse events and shall determine seriousness and causality (in conjunction with the medical practitioner steering group representative, if necessary).

## Discussion

To our knowledge, this is the first RCT of an occupational therapy intervention in a homecare re-ablement service setting. The Social Care Institute for Excellence (SCIE) in England has highlighted, as a research priority, the comparison of outcomes for users who receive occupational therapy treatment as part of their re-ablement period with those who do not [4]. This study will provide the foundations for a further, appropriately powered study to investigate this. Therefore the findings from this study will be relevant to researchers, clinicians, commissioners and service users. The findings will also assist the development and configuration of homecare re-ablement services in the future.

We plan to disseminate our findings through presentations at national and international rehabilitation and occupational therapy conferences, and will submit findings for publication in a peer-reviewed academic journal. This study also forms part of PW’s National Institute for Health Research (NIHR) funded PhD and the findings will also be written up as part of his thesis.

## Trial status

Recruitment commenced in April 2014. The trial is registered with Clinicaltrials.gov with the ISRCTN number 21710246 (registered on 31 March 14). Recruitment is expected to finish in November 2014.

## Abbreviations

ADL: Activities of daily living
ASCOT: Adult Social Care Outcomes Toolkit
BI: Barthel index
COT: College Of Occupational Therapists
CRF: Case report form
CSI: Caregiver Strain Index
GCP: Good clinical practice
NCTU: Nottingham Clinical Trials Unit
NEADL: Nottingham Extended Activities Of Daily Living
RCT: Randomized controlled trial
REC: Research Ethics Committee
SCIE: Social Care Institute For Excellence
SF-36: Short-form 36
TSC: Trial Steering Committee.
